# Generative Few-Shot Siamese Networks for Anomaly Detection: Application to Pipeline Leakage in Nuclear Power Plants

**DOI:** 10.3390/s26144372

**Published:** 2026-07-10

**Authors:** Jae-Hyeok Jeong, You-Rak Choi, Yong-Hoon Choi, Dong-Yun Cho, Min-Suk Kim

**Affiliations:** 1 Department of Electronic Information System Engineering, Sangmyung University, Cheonan 31066, Republic of Korea; 2023d1013@sangmyung.kr; 2 Nuclear System Integrity Sensing and Diagnosis Division, Korea Atomic Energy Research Institute, Daejeon 34057, Republic of Korea; yrchoi@kaeri.re.kr; 3 Department of Human Intelligence and Robot Engineering, Sangmyung University, Cheonan 31066, Republic of Korea; 2026d1006@sangmyung.kr; 4 Department of Nuclear System Engineering, University of Science and Technology (UST), Daejeon 34113, Republic of Korea; dycho@kaeri.re.kr

**Keywords:** SiameseGAD, siamese networks, few-shot anomaly detection, data generation, pipe leak, DDPM

## Abstract

In safety-critical industrial environments such as nuclear power plants (NPPs), early detection of pipeline leakage is essential for maintaining operational safety. However, leakage events are rare, abnormal samples are difficult to collect, and obtaining sufficient condition-specific normal data is also challenging. To address these limitations, this paper proposes SiameseGAD, a generative few-shot anomaly detection framework for pipeline leakage detection in the secondary systems of NPPs. The proposed method formulates leakage detection as a few-shot normality-modeling problem rather than as a problem of directly learning anomaly patterns. The Siamese network learns similarity relationships among normal samples and constructs a normal feature manifold, while anomaly scores are computed based on the distance from the estimated normal distribution. To improve normal distribution estimation under limited data, a denoising diffusion probabilistic model (DDPM) is used to generate in-distribution normal variants to augment the support-set. The main contribution of SiameseGAD lies in combining metric-learning-based few-shot normality modeling with normal-to-normal generative augmentation, enabling anomaly detection using only a few normal samples without relying on real anomaly data or synthetic anomaly generation. In the three evaluated target classes, SiameseGAD achieved an average AUROC of 93.59% and an average accuracy of 95.26%. These results indicate the potential of SiameseGAD for few-shot anomaly detection using only normal support samples, without requiring real or synthetically generated anomaly samples during inference.

## 1. Introduction

With industrial environments becoming increasingly complex and automated, there is a growing need for technologies that can ensure system safety in real time. In particular, in high-risk facilities such as nuclear power plants (NPPs), minor system anomalies can lead to loss of life and huge property damage. In such high-risk environments, the early identification of subtle deviations from normal operating conditions is essential. Among various types of anomalies, pipeline leaks in secondary systems are considered both practically important and challenging. Compared to primary systems, secondary systems operate under relatively less stringent safety requirements and involve complex and extensive piping networks, increasing the likelihood of leaks. Although such faults may not immediately lead to catastrophic consequences, undetected leaks can result in system efficiency degradation and equipment damage and may eventually escalate into more severe operational issues. Furthermore, leakage-related signals are typically weak and can be easily obscured by background noise and varying operating conditions, making early detection particularly challenging in real-world environments. In addition, safety constraints and rare occurrences of leakage events make it difficult to obtain sufficient defect data. Therefore, pipeline leak detection necessitates the adoption of anomaly detection methods that can operate effectively under few-shot learning scenarios.

Since supervised learning requires sufficient labeled anomaly data, most practical anomaly detection methods rely on unsupervised or self-supervised learning. In real-world environments, anomalies are rare and highly diverse, making them difficult to define clearly or collect systematically. Consequently, many anomaly detection systems adopt unsupervised or single-class learning paradigms that mainly use normal data to model normal states and detect deviations as anomalies. Unsupervised learning approaches to anomaly detection are broadly classified into reconstruction-based and embedding-based methods. Reconstruction-based methods employ models such as autoencoders to reconstruct normal data and identify anomalies based on the resulting reconstruction error. In contrast, embedding-based methods model the normal embeddings in the latent space and determine whether a sample is anomalous by measuring how far it deviates from this distribution. These approaches are widely applied in real-world environments since they can detect anomalies using only normal data for training. However, their use in high-risk environments remains an important challenge, and unsupervised learning methods still require large amounts of high-quality normal data to achieve strong performance. In real-world scenarios based on different types of anomalies and different operating environmental conditions, separate models are often required for each condition or category. In such cases, collecting sufficient representative normal data for every case is highly expensive and time-consuming. While simulation-based methods are being explored as potential solutions to these limitations, distribution discrepancies between simulated and real data often lead to significant performance degradation and poor generalization.

Recently, few-shot anomaly detection (FSAD) has gained increasing attention because it enables anomaly detection under conditions where only a limited number of samples are available. Existing FSAD approaches include meta-learning-based, metric-learning-based, and generative augmentation-based methods. Meta-learning-based methods follow a “learning-to-learn” paradigm and aim to rapidly adapt a model to a new task or operating condition using experience acquired from multiple training tasks [1,2,3,4]. In contrast, metric-learning-based methods, including Siamese networks [5] and prototypical networks [6], learn similarity or distance relationships between samples in an embedding space. These methods are suitable for limited-data settings because they can form discriminative feature representations using only a small number of support samples. GAD-PN [7] combines prototypical-network-based few-shot learning with CycleGAN-based anomaly generation [8], whereas RegAD [9] adopts a Siamese architecture to learn shared representations and estimate normality using limited normal samples from a target category. By learning pairwise relationships between input samples, Siamese networks are structurally advantageous for constructing meaningful representations with only a small number of samples. RegAD is designed to cluster representations of normal samples in a high-dimensional embedding space via a single loss function, enabling robust anomaly detection with limited normal data. Since anomalies such as pipe leak events are rare and labeled anomaly data are difficult to obtain in practical industrial environments, we consider an FSAD setting in which only a small number of normal samples are available in a target environment. This motivates the adoption of an FSAD framework for ultrasonic leak monitoring in high-risk environments.

In this paper, we propose SiameseGAD, a generative few-shot anomaly detection framework for pipeline leakage detection in the secondary systems of nuclear power plants. The proposed method is based on the Siamese-network-based FSAD structure of RegAD and constructs a normal feature manifold by learning similarity relationships among normal samples. During inference, the normal feature distribution is estimated using a small number of normal support samples provided under the target condition, and the anomaly score is calculated based on how far a query sample deviates from this distribution. Under few-shot conditions, however, estimation of the normal distribution may become unstable because the number of support samples is highly limited. To address this issue, SiameseGAD uses a DDPM-based generative model [10] to generate in-distribution variants of normal samples and employs them for support-set augmentation. The proposed DDPM-based augmentation is not intended to generate synthetic anomaly data; rather, it aims to complement normality modeling through normal-to-normal augmentation. In addition, this strategy is based on the assumption that normal characteristics are partially shared across different operating conditions or scenarios, and its effectiveness may be limited when there is a large distribution gap between the source and target conditions.

In addition, the proposed framework transforms signals collected from ultrasonic sensors into FFT-based frequency representations and then converts them into two-dimensional image representations using the Gramian Angular Difference Field (GADF). The GADF-based representation is used to express the frequency patterns and correlations contained in ultrasonic leakage signals as spatial structures that can be processed by CNNs. The proposed method is evaluated using ultrasonic leakage data organized into condition-specific dataset classes and collected from a simplified experimental setup related to leakage conditions in the secondary systems of nuclear power plants, demonstrating its applicability to data-scarce industrial anomaly detection. The main contributions of this paper are as follows:

- We propose SiameseGAD, an FSAD framework designed for pipeline leakage detection in the secondary systems of nuclear power plants, where real anomaly data and large-scale condition-specific normal data are difficult to obtain.
- SiameseGAD constructs a normal feature manifold through metric-learning-based few-shot normality modeling. Specifically, it learns similarity relationships among normal samples in an embedding space and computes anomaly scores based on deviations from the estimated normal feature distribution during inference.
- We introduce a DDPM-based generative augmentation strategy that generates in-distribution normal variants from limited normal samples. This improves normal distribution estimation under few-shot conditions without relying on real anomaly data or synthetic anomaly generation.
- The proposed method is evaluated using ultrasonic leakage scenario data collected from a simplified experimental setup related to leakage conditions in the secondary systems of nuclear power plants, demonstrating its applicability to data-scarce industrial anomaly detection.

## 2. Related Works

### 2.1. Traditional Anomaly Detection

Traditional anomaly detection approaches are primarily based on unsupervised learning, relying solely on normal data to capture the statistical and structural characteristics of normal patterns, and detecting as anomalies any data that deviates from these characteristics. These approaches are effective in real-world scenarios where anomaly data are scarce and are commonly divided into reconstruction-based and embedding-based methods. Reconstruction-based anomaly detection methods operate under the assumption that models trained exclusively on normal data can accurately reconstruct normal samples, but fail to reconstruct anomalies. Therefore, the difference between input and reconstruction is used as an anomaly score. Early approaches were mainly based on autoencoders and variational autoencoders (VAEs) [11,12], which typically reconstructed the entire input and analyzed the reconstruction error. However, these models can reconstruct anomalies with the same fidelity as normal data, which results in reduced detection accuracy. To overcome this limitation, several strategies such as image masking and pseudo-anomaly generation have been proposed. For example, DRAEM [13] integrates the reconstruction of pseudo-anomaly images into normal ones with a discriminator to perform anomaly localization. UniAD [14], on the other hand, improves reconstruction quality through a transformer-based architecture and additional enhancements.

In addition, the embedding-based approach estimates the distribution of normal data using feature embeddings extracted from pre-trained models and detects anomalies by measuring the extent to which test samples deviate from this distribution. Representative methods in this category, such as SPADE [15], PaDiM [16], and PatchCore [17], have demonstrated strong performance on industrial anomaly detection benchmarks such as MVTec [18]. However, their practical applicability is limited by the need for large amounts of high-quality normal data and the need to train separate models for each category.

### 2.2. Few-Shot Anomaly Detection

FSAD addresses the problem of detecting anomalies in new conditions or categories using only a limited number of samples, and it has attracted increasing attention as an important learning paradigm for industrial environments where data acquisition is difficult. Existing FSAD studies can be broadly categorized into meta-learning-based approaches, metric-learning-based approaches, and generative augmentation-based approaches. Meta-learning-based methods aim to rapidly adapt to new conditions by leveraging experience learned from diverse tasks or categories. For example, ref. [2] applied MAML-based meta-learning to rotating machinery data and demonstrated effective adaptation to new fault types using only a few data points. OC-MAML [19] performs meta-learning using only normal samples in each episode and rapidly forms decision boundaries for new classes during testing with a few normal samples. These methods provide rapid adaptation capability, but their performance can be affected by the similarity between meta-training tasks and target tasks, as well as by the configuration of episodic training.

Metric-learning-based FSAD methods learn distances or similarities between samples in the feature space to determine anomaly states under limited-sample conditions. Prototypical Network (PN)-based methods construct prototypes of normal and abnormal samples and determine whether a query sample is anomalous based on its distance to the prototypes. PCSNet [20] incorporates context-aware segmentation and pixel-level loss into the PN framework, enabling fine-grained prototype construction at the pixel level. PRN [21] combines a PN-based architecture with residual structures to capture local patch-level feature differences more precisely. Siamese-network-based methods can construct a comparable feature space even with a small number of samples by learning similarity relationships between sample pairs. However, metric-learning-based methods may still suffer from unstable estimation of the normal feature distribution or prototypes when the number of support samples is extremely limited.

Generative augmentation-based FSAD has also been developed to compensate for limited support samples by generating additional samples or variations. GRAPHCORE [22] performed FSAD by combining PatchCore and Graph Neural Networks (GNNs) with datasets augmented through rotation. TDG [23] generates images with diverse transformations and uses a patch-level discriminative structure to determine anomalies. Anomaly Diffusion [24] is the use of a diffusion model to generate realistic anomaly images in few-shot settings. These methods can enrich the feature space under limited-data conditions; however, their performance may be constrained if the generated samples do not sufficiently reflect the characteristics of target anomalies. In particular, for industrial leakage signals, where anomaly events are rare and can vary depending on physical conditions, synthetic anomaly generation may not fully capture the diversity and physical meaning of real anomaly patterns.

Recently, attempts have been made to improve FSAD performance by combining meta-learning or metric-learning structures with generative augmentation. GAD-PN [7] integrates a PN-based meta-learning framework with CycleGAN [8] to generate anomaly data from normal samples in the target domain, thereby alleviating the shortage of anomaly samples. RegAD [9] adopts Siamese networks to learn shared representations across classes and complements the feature space using normal samples from the target domain. In contrast, SiameseGAD generates normal-to-normal sample variations using DDPM instead of generating synthetic anomalies. This strategy aims to estimate the normal feature distribution more reliably using only a small number of normal support samples and is distinguished from previous generative FSAD methods in that it does not rely on anomaly generation. Therefore, SiameseGAD is designed to complement normality representation under few-shot conditions by combining metric-learning-based normality modeling with generative support-set augmentation.

### 2.3. Time-Series-to-Image Transformation

Time-series anomaly detection has traditionally been performed using sequence-learning or reconstruction-based models such as RNNs, LSTMs, and autoencoders. These methods have the advantage of directly learning temporal dynamics; however, their performance and computational cost can vary significantly depending on data characteristics, sequence length, training data size, and model architecture. Recently, studies have explored transforming time-series data into two-dimensional image representations to leverage the feature extraction capability of CNNs. The Gramian Angular Field (GAF) transforms time-series values into polar coordinates and represents temporal correlations in a matrix form, enabling temporal correlations to be expressed as spatial patterns. For example, studies that transformed electrocardiogram (ECG) signals into GAF representations reported improved performance in myocardial infarction detection [25]. GAF-based CNN models have also shown potential in applications such as fluid dynamics prediction and anomaly detection in manufacturing processes [26].

Additionally, condition monitoring under varying operating conditions has been investigated using condition-adaptive signal-analysis approaches, including statistic-discrepancy-oriented cyclo-non-stationary indicators [27] and slice-oriented signal probability distribution measures [28]. These approaches characterize condition-related distribution changes and mitigate the effects of dynamically varying operating environments. In contrast, SiameseGAD focuses on few-shot normality modeling by estimating the target normal feature distribution using a small number of normal support samples. SiameseGAD can adapt to previously unseen target conditions, but it does not explicitly model continuously varying operating conditions. Addressing such conditions remains an important direction for future research.

## 3. Piping Leak Dataset Description and Analysis

### 3.1. Pipeline Leakage Data Collection

Due to strict safety regulations in nuclear power plants, it is not feasible to reproduce abnormal situations. As a result, collecting sufficient leakage data from real facilities is limited, which makes the development and evaluation of data-driven anomaly detection models challenging. To alleviate this limitation, a laboratory-scale apparatus capable of controlled air leakage experiments was constructed based on the American Society for Testing and Materials (ASTM) E1002-11 standard [29]. The ASTM E1002-11-based experimental procedure provides consistent data collection conditions for ultrasonic leak testing. However, the apparatus was not intended to fully reproduce all physical and operational conditions of an actual nuclear power plant. Instead, it was designed as a laboratory-scale setup to simulate simplified conditions related to pipeline leakage in the secondary systems of nuclear power plants.

**Figure 1:**
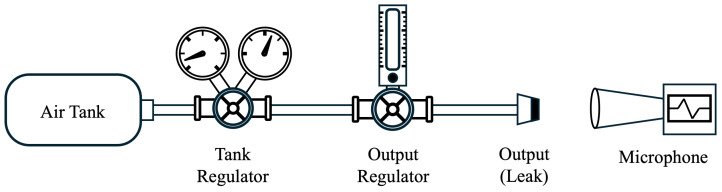
Experimental setup for data collection in pipeline leakage detection.

Figure 1 illustrates the experimental setup and Table 1 summarizes the data collection conditions. Ultrasonic signals in the range of 20 kHz to 100 kHz were collected using the low-power wireless ultrasonic sensor module proposed in [30]. Because ultrasonic signals attenuate with increasing propagation distance, the analog signals captured by a microphone were first amplified using an amplifier and then digitized via an analog-to-digital (A/D) converter with a sampling frequency of 256 kHz. The digitized signals were transformed into the frequency domain using the fast Fourier Transform (FFT) according to the data processing procedure of the previous work [7]. Subsequently, frequency components in the 20–100 kHz range were extracted at 0.25 kHz intervals, and the averaged spectrum of each signal was represented as a fixed-length 320-dimensional spectral vector.

### 3.2. Analysis of the Dataset Classes

The experimental datasets, covering a range of leak conditions, were constructed following the experimental protocol of the previous work [7]. Using the ultrasonic data acquisition system described in Section 3.1, the dataset was organized into four condition-specific classes, denoted as A, B, C, and D. Each class was designed to represent a distinct leakage-related measurement condition and contained samples from both normal and leakage states. These classes were not intended to reproduce the full range of operating environments in actual nuclear power plants. Rather, they were constructed as simplified experimental conditions related to pipeline leakage in the secondary systems of nuclear power plants. Therefore, the dataset should be interpreted not as a complete substitute for real field data but as an experimental dataset for analyzing differences between normal and leakage ultrasonic signals and evaluating few-shot anomaly detection models under controlled conditions.

**Figure 2:**
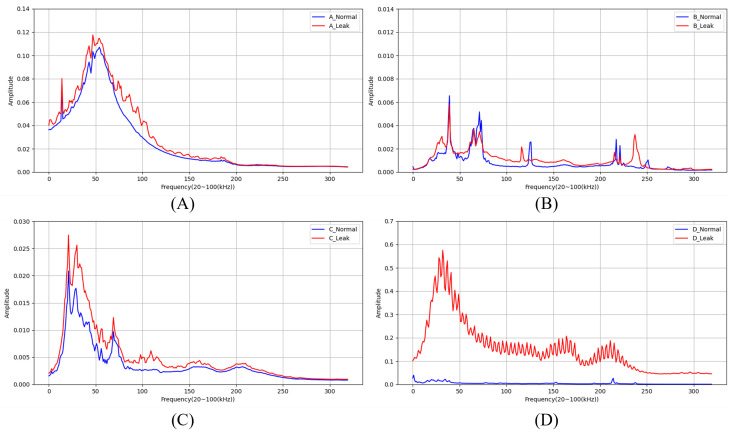
Averaged ultrasonic spectra comparing normal and leak conditions: (**A**) Domain A; (**B**) Domain B; (**C**) Domain C; (**D**) Domain D.

The collected data were stored separately for normal and leakage states and include time-domain, frequency-domain, and ultrasonic-specific features. Their statistical characteristics and periodic patterns were analyzed using the autocorrelation function, correlation analysis, and time-varying pattern analysis. The final dataset consists of both normal and leakage samples and was used for model training as well as evaluation. Figure 2 illustrates representative normal (blue) and leakage (red) signals for each of the four dataset classes, ordered from left to right as A, B, C, and D. The number of samples in each dataset class is summarized in Table 2.

## 4. Materials and Methods

### 4.1. Frequency-to-2D Image Conversion via GADF Transformation

Transforming time-series or sensor signals into two-dimensional image representations has been used as an effective approach to leverage CNN-based feature extraction. CNNs can hierarchically learn local patterns within input data and are useful for capturing repetitive or localized patterns in structured two-dimensional representations. However, image-based transformation is not universally superior to all time-series learning methods. Therefore, in this study, GADF transformation is not considered a universally optimal representation method; rather, it is used to represent the frequency patterns and correlations of ultrasonic leakage signals as spatial structures that can be processed by CNNs.

**Figure 3:**
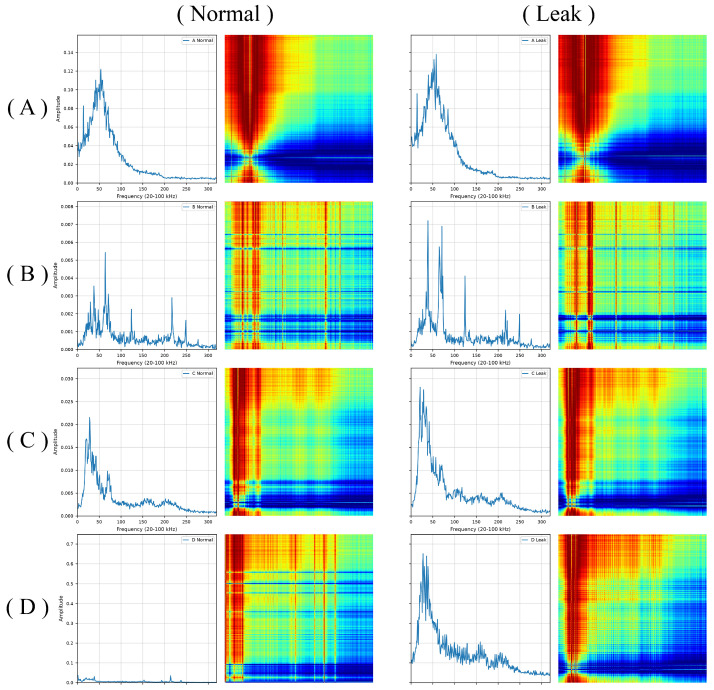
Representative examples of GADF-transformed images: (**A**) Domain A; (**B**) Domain B; (**C**) Domain C; (**D**) Domain D.

In this paper, we use a GADF-based transformation to convert ultrasonic signals into image-based representations, following the approach used in GAD-PN [7]. Specifically, the original ultrasonic signals are first transformed into the frequency domain using FFT and then encoded into two-dimensional images through a GADF-based transformation. GAF normalizes time-series values, maps them into a polar coordinate system, and then applies trigonometric operations to represent relationships between temporal or frequency components as visual patterns in the form of square matrices. Depending on the transformation method, the Gramian Angular Summation Field (GASF) captures summation information between intervals, whereas the Gramian Angular Difference Field (GADF) emphasizes differences and variation patterns between components. In this study, we use the GADF transformation, as shown in Figure 3, to emphasize variation information in the frequency-domain patterns of ultrasonic leakage signals [31]. However, because GADF transformation does not preserve all fine-grained temporal information of the raw signal, it is interpreted not as a superior alternative to raw-sequence learning but as a preprocessing method for converting frequency-based leakage patterns into structured representations suitable for CNN input.

### 4.2. Siamese Network Architecture for Few-Shot Anomaly Detection

**Figure 4:**
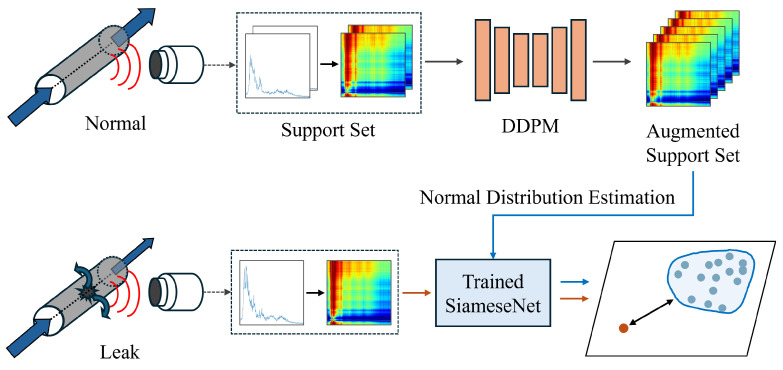
Overview of the SiameseGAD workflow for pipeline leakage detection.

In this paper, we propose a metric-learning-based anomaly detection framework that adapts the Siamese architecture of RegAD [9] to ultrasonic pipeline leakage data. The input data are constructed from 320-dimensional fixed-length spectral vectors extracted from the 20–100 kHz frequency range at 0.25 kHz intervals. These spectral vectors are then encoded into GADF images for Siamese-network-based feature learning. The GADF images are generated by transforming numerical ultrasonic signals, where each pixel represents a fixed relationship between transformed components. In RegAD, geometric transformations such as rotation, translation, and scaling are used to generate pseudo-anomalies that mimic real defects in industrial products. In our case, however, the data are represented by time-frequency patterns encoded by GADF corresponding to fixed time and frequency relationships. Thus, applying such geometric transformations does not realistically reflect real-world anomalies in the data but rather distorts the intrinsic temporal and frequency structures. Moreover, these augmentation strategies are not suitable for simulating anomalies in our setting. Because geometric registration is not physically meaningful for these frequency-domain representations, the STN modules are omitted from the proposed framework. Furthermore, to improve adaptability to the proposed dataset, we replace the existing image augmentation strategies used in RegAD for normal-distribution estimation at test time with a generative model-based approach. Our method employs a pretrained backbone and data from the source classes during training, allowing a single model to generalize over multiple classes and rapidly adapt to a previously unseen target class using only a few normal support samples. An overview of the proposed framework is shown in Figure 4.

#### 4.2.1. Siamese Feature Representation Learning

**Figure 5:**
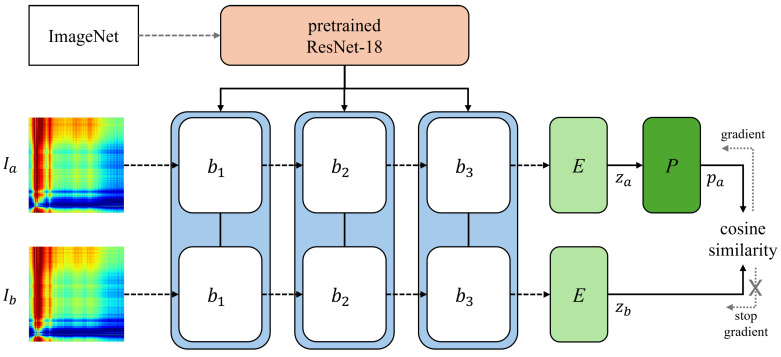
Training framework of the SiameseGAD.

As shown in Figure 5, the proposed model takes as input an image pair consisting of two normal images, $I_{a}$, $I_{b}$, and learns the relative feature similarity between the two images. Similar to RegAD, the backbone is built from the initial three convolutional blocks of ResNet-18, which have been pretrained on the ImageNet dataset. The two input images $I_{a}$ and $I_{b}$ pass through the same encoder E(·) and are transformed into feature projections $z_{a}=E\left(b_{3}\left(I_{a}\right)\right)$ and $z_{b}=E\left(b_{3}\left(I_{b}\right)\right)$, respectively. The predictor P(·) then maps each projection to $p_{a}=P\left(z_{a}\right)$. Following the SimSiam [32] strategy, the predictor output from one branch is compared with the stop-gradient projection from the other branch, allowing the model to learn similarity relationships among normal samples while preventing feature collapse without negative pairs.

The loss function is defined based on the negative cosine similarity between the two embeddings, as given by the following Equation (1).

(1) $$D\left(p_{a},z_{b}\right)=-\frac{p_{a}}{∥p_{a}{∥}_{2}}\cdot \frac{z_{b}}{∥z_{b}{∥}_{2}}$$

The final loss is formulated as a bidirectional average, as shown in Equation (2).

(2) $$L=\frac{1}{2}\left(D\left(p_{a},z_{b}\right)+D\left(p_{b},z_{a}\right)\right)$$

This architecture enables the formation of consistently comparable feature spaces across multiple categories while preventing feature collapse without negative pairs. Furthermore, considering the nature of the dataset used in this paper, the omission of STN modules can efficiently reduce network complexity and save computational resources.

#### 4.2.2. Quantitative Anomaly Score Estimation

During the testing phase, the trained Siamese network remains fixed and estimates the normal feature distribution using a support set consisting of a limited number of normal samples from the new category. Each support image is augmented using a generative model, and the feature set at each patch location is modeled as a multivariate normal distribution $N(\mu_{ij},\Sigma_{ij})$. The location-specific feature $f_{ij}$ is extracted from the test image, and the anomaly score is calculated using the Mahalanobis distance, as shown in Equation (3).

(3) $$M\left(f_{ij}\right)=\sqrt{{\left(f_{ij}-\mu_{ij}\right)}^{⊤}\Sigma_{ij}^{-1}\left(f_{ij}-\mu_{ij}\right)}$$

### 4.3. DDPM-Based Normal Data Augmentation

In Siamese-network-based FSAD, data augmentation is commonly used to expand the support set via label-preserving transformations (e.g., rotation, translation, flipping, and grayscale conversion). However, the input is a frequency-domain GADF image derived from an ultrasonic signal, where pixel positions encode fixed time-frequency relationships rather than object geometry in our proposed method. Conventional affine transformations, such as rotation and translation, can interfere with basic time-frequency semantics and degrade detection performance. Moreover, GAN-based augmentation can be unstable if the number of training samples available in few-shot settings is limited. Based on these considerations, we adopt a diffusion-based generative augmentation strategy to generate meaningful variations while preserving the underlying structure of frequency images.

**Figure 6:**
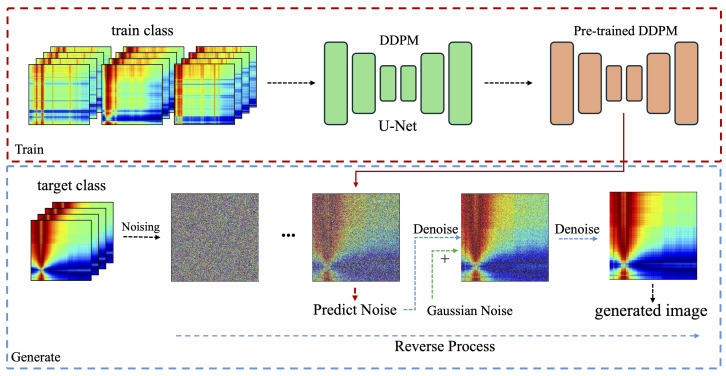
Workflow of the augmented data generation process using DDPM.

DDPM is a stochastic generative model designed to approximate complex distributions of high-dimensional data. The data distribution is learned through a forward process, and it gradually adds Gaussian noise to the input data according to the diffusion schedule, eventually converting it into standard Gaussian noise. A reverse process then progressively removes the noise to reconstruct the original data. Through this two-step process, the model learns to generate data that retains the essential characteristics of the training distribution. In this paper, synthetic normal samples are generated by injecting noise into normal frequency-image data and applying the reverse process of DDPM. The generated images are incorporated into the support-set during the few-shot learning process and are used to complement the normal feature distribution estimated from limited normal samples. More specifically, the DDPM is trained using normal frequency-image data from the source classes, excluding the target class selected for anomaly detection. Gaussian noise is then applied to normal support images from the target class, and the trained DDPM reverse process is used to generate normal variants aligned with the learned normal distribution. The generated images are incorporated into the support-set during the few-shot learning stage, enriching the representation of the normal distribution and enhancing anomaly detection performance. An illustration of this process is provided in Figure 6.

## 5. Experiments and Results

### 5.1. Experimental Setup

For the diffusion-based generative model, we used an AdamW optimizer with a learning rate of 3 × 10^−4^. The generative process was performed using 1000 diffusion steps to enhance data diversity, and the beta schedule was linearly set from 1 × 10^−4^ to 0.02. In the SiameseGAD framework, a stochastic gradient descent (SGD) with a learning rate of 0.0001 and momentum of 0.9 was used to ensure stable convergence during training. All training and inference experiments were conducted on a dedicated workstation running Ubuntu 20.04, equipped with an NVIDIA RTX A6000 GPU, an AMD Ryzen Threadripper PRO 3955WX 16-core CPU, and 16 GB of RAM.

### 5.2. Results of Normal Data Generation

**Figure 7:**
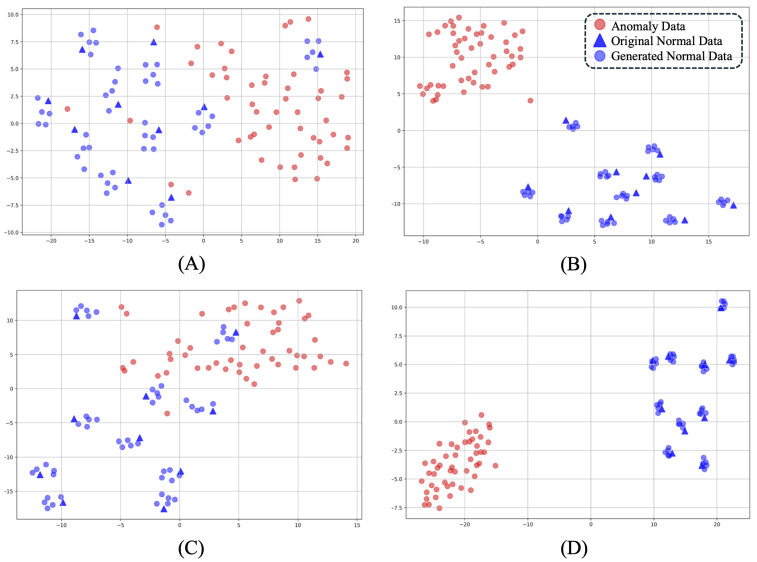
t-SNE visualization of original and DDPM-generated normal samples for each domain: (**A**) Domain A; (**B**) Domain B; (**C**) Domain C; (**D**) Domain D.

In this paper, normal samples are generated using DDPM for support-set augmentation, and their effectiveness is validated by visually analyzing the similarity in the embedding space between the generated and original normal samples. For this purpose, t-SNE is applied to examine how closely the generated samples conform to the distribution of actual normal data. Furthermore, within the assumed few-shot setting, sample generation for a specific class is carried out using a DDPM model trained exclusively on data from all other classes, excluding the target class. This process is shown in Figure 7.

Figure 7 presents t-SNE visualizations of the embedding space. For each dataset class, 10 normal samples were randomly selected, and five synthetic variants were generated for each sample using the DDPM. DDPM-generated samples were located close to the corresponding original normal samples in the embedding space, suggesting that the generated samples preserved embedding-level normal characteristics. The purpose of DDPM-based generation is not to produce physically complete normal samples but to augment the support set and improve normal feature distribution estimation under few-shot conditions. These observations suggest that DDPM-based augmentation can increase the diversity of the support set while preserving the principal characteristics of normal samples.

### 5.3. Comparative Evaluation: RegAD, RegGAD, and SiameseGAD

To evaluate the contribution of each architectural component, we compared SiameseGAD with two structural variants of RegAD. In RegAD, data augmentation is primarily performed using conventional techniques such as rotation and translation. However, in this paper, the input data consist of frequency-based images derived from time-series signals using FFT and GADF, for which traditional augmentation methods were found to be unsuitable, as they failed to preserve meaningful patterns after transformation. To overcome this limitation, we used a DDPM-based generative augmentation strategy capable of producing diverse, high-quality samples from a small set of normal instances. During inference, 10 new samples were generated for each input normal instance to facilitate a more accurate estimation of the normal data distribution. In addition, although RegAD contains STN modules for geometric alignment, these alignments can be irrelevant or potentially detrimental when applied to GADF-based frequency images where spatial distortion has no meaningful effect on performance. Therefore, STN modules were excluded in this study, and the framework consisted only of Siamese networks and Mahalanobis distance-based anomaly detection.

To further validate the contribution of each component in the proposed framework, we conducted an ablation study by comparing RegAD, RegGAD, and SiameseGAD. RegAD represents the original Siamese-network-based architecture using conventional augmentation, RegGAD replaces conventional augmentation with DDPM-based generative support-set augmentation, and SiameseGAD further removes the STN modules to better fit GADF-based ultrasonic frequency images. This comparison allows us to examine the impact of DDPM-based normal augmentation and architectural simplification on the performance of few-shot anomaly detection.

In addition, experiments were conducted over 100 fixed episodes. In each episode, 5 normal samples were randomly selected to form the support set, while the query set consisted of 15 normal and 15 abnormal samples randomly sampled for evaluation. Under this few-shot setting, SiameseGAD uses the limited normal samples of the support set to estimate the normal feature distribution and evaluates the remaining normal and abnormal query samples accordingly. Performance was evaluated with the Area Under the ROC Curve (AUROC), obtained by integrating the ROC curve defined by the true positive rate and false positive rate across thresholds. A value closer to 1 signifies stronger anomaly detection performance, and AUROC is widely recognized as a robust evaluation metric, particularly in scenarios with class imbalance.

As shown in Table 3, after replacing conventional augmentation with DDPM-based support-set augmentation, RegGAD yielded higher AUROC point estimates than RegAD in all three evaluated target classes. SiameseGAD yielded similar or slightly higher point estimates than RegGAD after the STN module was removed. These observations suggest that DDPM-based support-set augmentation and architectural simplification may positively contribute to performance under the evaluated conditions.

Table 3 presents a component-wise ablation and trend analysis under the same fixed experimental protocol. Therefore, the results are intended to examine the relative contributions of DDPM-based support-set augmentation and STN removal rather than to establish statistical superiority among the evaluated configurations. Episode-level variability for SiameseGAD and the external baselines is reported separately in Table 4 as the mean ± standard deviation over 100 episodes.

### 5.4. Experimental Results and Comparative Analysis of Few-Shot Anomaly Detection

To evaluate the FSAD performance of SiameseGAD, we compared its AUROC with those of PatchCore [17] and RealNet [33], under the same 100-episode evaluation protocol described in Section 5.3. In each episode, five normal samples were randomly selected as the support set, and the query set consisted of 15 normal and 15 abnormal samples. Accuracy was also descriptively compared with the results reported for GAD-PN [7]. Following its original evaluation protocol, GAD-PN used five normal and five abnormal support samples. In contrast, SiameseGAD used only five normal support samples, while abnormal samples were used exclusively for evaluation. Therefore, the accuracy comparison with GAD-PN should be interpreted as a reference comparison rather than as a direct statistical comparison under identical experimental conditions. The anomaly scores were min–max-normalized using the bounds estimated from the training data, and the same fixed threshold of 0.5 was applied to all target classes.

As shown in Table 4, the results for PatchCore, RealNet, and SiameseGAD are reported as the mean ± standard deviation over 100 episodes for each target class. Across the three evaluated target classes, the arithmetic mean of the class-specific AUROC values was 93.59% for SiameseGAD, compared with 64.97% for PatchCore and 60.19% for RealNet. Thus, SiameseGAD achieved average AUROC improvements of 28.62 and 33.40 percentage points over PatchCore and RealNet. The arithmetic mean of the class-specific accuracy values for SiameseGAD was 95.26%. The mean of the corresponding accuracy values for GAD-PN was 93.22%, showing a difference of 2.04 percentage points. However, since the two methods use different support-set configurations, this difference should be interpreted cautiously. The class-specific mean and standard deviation values are presented in Table 4.

Table 5 presents the comparison of the average inference times for each model, including the proposed SiameseGAD. RealNet achieved the fastest performance, requiring 10 GB of GPU memory and 0.01 s per image. GAD-PN used 10 GB and recorded the second-fastest inference time of 0.083 s. PatchCore consumed 11 GB with an inference time of 0.41 s, while the proposed SiameseGAD used 12 GB and achieved a comparable time of 0.42 s. Although SiameseGAD is slightly slower than RealNet and GAD-PN, it demonstrates an inference speed nearly identical to that of PatchCore.

Furthermore, we compared training configurations using two and three source classes to assess the effect of the number of source classes on model performance. As summarized in Table 6, increasing the number of source classes generally improved AUROC under the same target-class evaluation conditions. For instance, when class A was used for testing, training with classes B and C resulted in an AUROC of 87.28%, whereas adding class D (i.e., training with B, C, and D) improved the AUROC to 88.23%, an improvement of 0.95 percentage points. Similarly, the AUROC increased from 99.17% to 99.90% for class B and from 94.31% to 96.43% for class C, representing improvements of 0.73 and 2.12 percentage points, respectively. These results suggest that increasing the number of source classes enables the model to learn more diverse normal representations, thereby improving anomaly detection performance for previously unseen target classes. Despite the effectiveness of the proposed DDPM-based augmentation strategy, several limitations should be noted. Since the diffusion model is trained on normal data from non-target classes, the generated variants may not fully capture target-specific normal patterns, which can lead to a distribution mismatch when used for test-time augmentation. In addition, in ultra-few-shot settings, the diversity of synthesized samples can be insufficient to reliably expand the support set and stabilize estimation of the normal feature distribution. These limitations motivate future work on target-aware generative constraints and more robust augmentation strategies, especially when source and target classes exhibit substantial distributional differences.

## 6. Conclusions

In this paper, we proposed SiameseGAD, a few-shot anomaly detection method tailored to safety-critical environments such as nuclear power plant piping systems. Conventional anomaly detection methods typically require large-scale representative normal data or separate models for different operating conditions, which limits their practicality in real-world deployments. To address these challenges, SiameseGAD combines a Siamese-network-based normality modeling framework with DDPM-based normal-to-normal augmentation, enabling anomaly detection using only a small number of normal support samples.

The proposed model was evaluated on three target dataset classes using AUROC and accuracy as the evaluation metrics. Across the evaluated target classes, SiameseGAD achieved a higher average AUROC than the RegAD, PatchCore, and RealNet baselines. It also achieved a higher average accuracy than the reported GAD-PN results while using only normal support samples during inference. Because GAD-PN and SiameseGAD use different support-set compositions, this accuracy comparison should be interpreted as a reference comparison rather than as evidence of direct statistical superiority.

From a practical deployment perspective, the DDPM training process entails additional computational costs; however, these costs are incurred offline as a one-time pretraining phase. Once deployed, SiameseGAD performs anomaly inference based on the estimated normal feature distribution without repeatedly training the generative model during inference. This offline training and fixed-inference structure suggests potential applicability to industrial monitoring systems that require stable inference, although quantitative real-time deployment analysis remains for future work.

Nevertheless, several limitations remain to be addressed in future research. Since the DDPM is trained on normal data collected from the source classes, its effectiveness depends on how well normal characteristics are shared between the source and target classes. If the corresponding source and target distributions differ substantially, the generated normal variants may not fully represent the target-class-specific normal distribution. In addition, although SiameseGAD can adapt to a previously unseen target class using a few normal support samples, directly applying the current framework to deployment environments with continuously varying operating conditions may remain challenging. This is because the proposed method estimates the normal feature distribution from a fixed support set rather than explicitly modeling time-varying operating conditions. Future work will investigate target-aware generative modeling, adaptive support-set updating, and online adaptation strategies to improve robustness under continuously varying operating conditions.

## Figures and Tables

**Table 1:** Experimental standard and data collection setup for pipeline leakage detection.

Standard	Class	Condition	Value
ASTM E1002-11	Class II	Orifice diameter	≤0.2 mm
		Distance	5 m
		Pressure difference	0.7 atm
		Frequency	20∼100 kHz
		Leak rate	0.024 gpm

**Table 2:** Number of normal and leakage samples in each dataset class.

State	A	B	C	D
Normal	981	363	995	982
Leak	981	778	1149	1010

**Table 3:** Performance comparison between the proposed method and RegAD.

Model	Source Classes	Support Class	Target Class	AUROC (%)
RegAD	B, C	A	A	86.82
	C, A	B	B	97.38
	A, B	C	C	91.04
RegGAD	B, C	A	A	87.09
	C, A	B	B	99.02
	A, B	C	C	94.05
SiameseGAD	B, C	A	A	87.28
	C, A	B	B	99.17
	A, B	C	C	94.31

**Table 4:** Evaluation results of few-shot anomaly detection.

Model	Training Class	Support Class	Target Class	AUROC (%, Mean ± Std)	Accuracy (%, Mean ± Std)
PatchCore	A	.	A	56.13±1.01	
	B	.	B	78.27±0.74	
	C	.	C	60.51±1.26	
RealNet	A	.	A	58.92±2.12	
	B	.	B	60.45±0.78	
	C	.	C	61.19±1.32	
GAD-PN	B, C	A	A		91.43
	C, A	B	B		93.76
	A, B	C	C		94.46
SiameseGAD	B, C	A	A	87.28±1.58	92.08±1.44
	C, A	B	B	99.17±0.94	98.58±0.76
	A, B	C	C	94.31±1.68	95.13±1.30

**Table 5:** Evaluation results of mean inference time per image.

**Model**	PatchCore	RealNet	GAD-PN	SiameseGAD
**Time(s)**	0.41	0.01	0.083	0.42

**Table 6:** AUROC comparison according to the number of source classes.

Model	Train	Support Train	Test	AUROC (%)
SiameseGAD	B, C	A	A	87.28
	C, A	B	B	99.17
	A, B	C	C	94.31
SiameseGAD	B, C, D	A	A	88.23
	C, D, A	B	B	99.90
	A, B, D	C	C	96.43

## Data Availability

The data presented in this study are available from the corresponding author upon reasonable request.
